# Feasibility of randomized controlled trials and long-term implementation of interventions: Insights from a qualitative process evaluation of the PEDAL trial

**DOI:** 10.3389/fresc.2023.1100084

**Published:** 2023-02-01

**Authors:** Cathy Bulley, Pelagia Koufaki, Jamie Hugo Macdonald, Iain C. Macdougall, Thomas H. Mercer, Jane Scullion, Sharlene A. Greenwood

**Affiliations:** ^1^ School of Health Sciences, Queen Margaret University, Musselburgh, United Kingdom; ^2^ Institute for Applied Human Physiology, Bangor University, Bangor, United Kingdom; ^3^ Department of Renal Medicine, King’s College Hospital NHS Trust, London, United Kingdom; ^4^ Renal Medicine and Therapies, King’s College Hospital NHS Trust, London, United Kingdom; ^5^ Renal Sciences, Faculty of Life Sciences and Medicine, King’s College London, London, United Kingdom

**Keywords:** implementation, feasibility, sustainability, chronic kidney disease, hemodialysis, exercise, quality of life

## Abstract

**Introduction:**

A multi-site randomized controlled trial was carried out between 2015 and 2019 to evaluate the impacts on quality of life of an intradialytic exercise programme for people living with chronic kidney disease. This included a qualitative process evaluation which gave valuable insights in relation to feasibility of the trial and of the intervention in the long-term. These can inform future clinical Trial design and evaluation studies.

**Methods:**

A constructivist phenomenological approach underpinned face-to-face, semi-structured interviews. Purposive recruitment ensured inclusion of participants in different arms of the PEDAL Trial, providers with different roles and trial team members from seven Renal Units in five study regions. Following ethical review, those willing took part in one interview in the Renal Unit. Audio-recorded interviews were transcribed (intelligent verbatim) and inductively thematically analyzed.

**Results:**

Participants (*n* = 65) (Intervention arm: 26% completed; 13% who did not; Usual care arm: 13%; 46% women; 54% men; mean age 60 year) and providers (*n* = 39) were interviewed (23% PEDAL Trial team members). Three themes emerged: (1) Implementing the Intervention; (2) Implementing the trial; and (3) Engagement of the clinical team. Explanatory theory named “the Ideal Scenario” was developed, illustrating complex interactions between different aspects of intervention and trial implementation with the clinical context. This describes characteristics likely to optimize trial feasibility and intervention sustainability in the long-term. Key aspects of this relate to careful integration of the trial within the clinical context to optimize promotion of the trial in the short-term and engagement and ownership in the long-term. Strong leadership in both the clinical and trial teams is crucial to ensure a proactive and empowering culture.

**Conclusion:**

Novel explanatory theory is proposed with relevance for Implementation Science. The “Ideal Scenario” is provided to guide trialists in pre-emptive and ongoing risk analysis relating to trial feasibility and long-term intervention implementation. Alternative study designs should be explored to minimize the research-to-practice gap and optimize the likelihood of informative findings and long-term implementation. These might include Realist Randomized Controlled Trials and Hybrid Effectiveness-Implementation studies.

## 1. Introduction

Implementation research aims to address the research-to-practice time lag and the factors influencing this. Curran and colleagues (1) argue that as well as barriers relating to people, organizations and cultures, this delay is influenced by the stepwise approach to researching clinical efficacy, then establishing effectiveness, and finally researching implementation. Delaying research into external validity and implementation may miss opportunities both to optimize rehabilitation intervention acceptability and increase likelihood of long-term implementation. Reflection on the traditional research journey is crucial to ensure learning from experience alongside consideration of alternative research designs and methods that may be more supportive of long-term implementation.

In this paper, interactions between characteristics of effectiveness evaluation and potential for long-term implementation are explored using data from a specific clinical rehabilitation intervention and context: exercise during hemodialysis for people living with end-stage kidney failure. More than 21,000 people in the UK receive hemodialysis to manage their condition (2), however, longer life expectancy is not always accompanied by good quality of life (3). Numerous systematic reviews suggest that exercise training interventions can increase exercise capacity and physical function (4–17). Preliminary evidence suggests these improvements can also impact positively on quality of life, which is linked to reductions in mortality and morbidity (3, 18).

Evidence relating to exercise for people receiving hemodialysis consisted of small Trials, most of which were not considered to be of a high quality (11). Progression was needed through the development of a methodologically robust and adequately powered randomized controlled trial (RCT). Consequently, the PEDAL (“PrEscription of intraDialytic exercise to improve quAlity of Life in patients with chronic kidney disease”) Trial was conducted between 2015 and 2019 to evaluate the clinical effectiveness and cost-effectiveness of a six-month intradialytic exercise programme when compared with usual hemodialytic care (19). This multi-center RCT, with qualitative sub-study and process evaluation, focused on quality of life as a primary outcome. Participants were people living with end-stage kidney disease who had been receiving maintenance hemodialysis therapy for over a year. People from dialysis centers in five regions across the UK were web-randomized (random generation of treatment allocation carried out online) by the study Clinical Trials Unit to either usual care during hemodialysis (all aspects of hemodialysis treatment received normally with no additional intervention) or to the addition of exercise using static cycle ergometers (exercise bikes) during their thrice weekly dialysis sessions. Of 335 people who completed baseline assessments, 243 completed the follow-up data collection at six months. Analysis demonstrated that there was no statistically significant improvement in the primary outcome in the intervention group compared with the usual care group (*p *=* 0.055*). The study team concluded that the intervention did not effectively improve quality of life in this context.

The qualitative sub-study aimed to explain the impacts of the intervention when people were able to sustain participation and summarize influences on both trial and intervention participation throughout the study. This is explained in the full published study report in greater detail (19). Barriers to participation were found to be both individual (e.g., health status) and contextual, with influences from the Renal Unit as an integrated community and culture. Participants needed support at multiple levels to engage in the trial and continue participation in the intervention. For people who continued exercising throughout the study, qualitative data described physical, psychological, functional and social benefits and improved quality of life (19). This conclusion contrasted with the findings of the primary quantitative analysis but aligned with the multiple barriers to maintaining participation. Analysis indicated that people were mutually influential in relation to the study and intervention and these interdependencies were affected by leadership and culture.

The in-depth analysis generated further original insights of value to people planning a RCT within a community of people who are providing and receiving a similar service with rehabilitation elements over the long-term. The insights are particularly nuanced due to the collection of substantial amounts of qualitative data from people with different roles in and experiences of the trial, and from multiple contexts across the UK. Therefore, this qualitative analysis addressed the questions: what were the key insights gained in relation to the feasibility of the trial and sustainability of the intervention in the long-term? How can these insights inform future rehabilitation intervention evaluation studies with a view to implementation? The original study aim was to explore expectations and experiences of exercise training in people experiencing the PEDAL Trial as participants and as providers within the Renal Unit and the study team and it was possible to address the further research questions through these data.

## 2. Materials and methods

Ethical approval was granted by London Fulham Research Ethics Committee (reference 14/LO/1,851) and informed consent was provided by all participants.

A constructivist phenomenological approach (20) was used in conducting and analyzing data from face-to-face, semi-structured interviews. Participants were recruited from Hemodialysis Units in each of five regions: London (two sites); Central Scotland (two sites); North Wales and North-West (one site); East Midlands (one site) and West Midlands (one site). Participants were purposively recruited to ensure representation of people who had been randomized to: the intervention arm of the study and completed the intervention; the intervention arm of the study but dropped out of the intervention and remained within the trial; and the usual care arm of the study. Providers were recruited to include people involved with or employed by the PEDAL Trial and people working within the Renal Unit in different roles.

People were given an invitation and information letter by the person delivering the intervention at each site. They were able to ask questions and, if willing, signed a consent form before being interviewed once when available in the Renal unit. Semi-structured topic guides were used which focused on the study aims and were reviewed by the PEDAL project team and the Ethics Committee and pilot tested to ensure questions flowed and were understandable (Topic guides with all questions are included in Supplementary File S1).

Most interviews were conducted by CB (Academic with physiotherapy undergraduate training and post-doctoral level qualitative research expertise) who had responsibility for leading the qualitative sub-study and was not involved in other aspects of trial implementation, with no prior relationships with any trial participants and most of the providers interviewed. She trained a research assistant (Academic with Masters level qualification) who had no other involvement in the study and supervised her in conducting a minority of interviews. CB introduced herself to interviewees and explained that within this type of qualitative research her aim was to represent their views and experiences and not her own. CB was not involved with the rest of the trial or intervention and wished to hear the person's views and experiences in their own words. CB emphasized that participants' identities and data would be protected. People could move on from any question or end the interview at any time and were informed that interviews would not take more than one hour. Interviews took place when and where the interviewee preferred and all wished to be interviewed at the Renal Unit, despite occasional interruptions from other people within the unit. All interviews were individual except for two people who regularly received hemodialysis in a side room and requested a joint interview. Each site started and progressed recruitment at different times, resulting in interviews being carried out over 16 months, at different points in each participant's trial journey (May 2016 – September 2017). Analysis of participant characteristics took place when the study was unblinded in 2019.

Audio-recorded interviews were transcribed (intelligent verbatim) and anonymized. Participant verification of interview summaries was planned into the study; a lack of response from participants in the first two of five regions (large geographical regions within the UK) led a decision not to continue with this as the impact was believed to be insufficient. Field notes were kept by the interviewers and key points were integrated into transcripts prior to analysis.

Inductive thematic analysis (20) was carried out by CB and JS, supported by NVivo v10. Inductive thematic analysis is consistent with the constructivist phenomenological research approach taken. It enables findings to emerge from what is said by the interviewees in an inductive manner while ensuring that there is an audit trail in relation to decision-making and evidence for themes, increasing rigour. Key ideas were noted on transcripts while reading and re-reading and organized both in NVivo and in Mindjet MindManager 2019. These were synthesized to form sub-themes that linked similar ideas. Sub-themes were grouped based on defined conceptual similarities, forming themes. Themes and sub-themes were given codes to support data management and provide an audit trail. The terminology of “themes” and “sub-themes” was used to describe groupings of text with similar meaning. The term “code” was used to indicate the location of themes and sub-themes in relation to one another (e.g., Theme 1 and sub-theme 1a). This process was carried out for each transcript, leading to a final thematic framework, which was then re-applied across all transcripts by JS. Where participants connected ideas as they talked, this was noted (for example, descriptions of people ending their participation in the study because they were invested in the intervention but were allocated to the usual care group). These connections provided evidence of linkages between themes which informed development of explanatory theory relating to the study data, illustrated diagrammatically (CB). Explanatory theory was developed to enable conclusions about why specific findings emerged, such as why some people may have had more positive experiences of participation in the trial than others. Explanatory theory can help to inform guidance about how things can be optimized in the future. Finally, analysis explored how themes differed between site and region. It is not possible to share the full transcript data as consent was not obtained for this at the outset and the risk of identification from combined data in transcripts is too great. Instead, the research team has made the in-depth mind maps available as supplementary files to illustrate the process of increasing abstraction (See Supplementary Files S2, S3).

## 3. Results

### 3.1. Participants

Interviews were completed with 22% of the participants who were remained in the PEDAL Trial at six months (*n* = 65). By region, this included 16 people from London, 22 from Central Scotland, seven from North Wales and North-West, 11 from East Midlands, and nine from West Midlands. The intention had been to include 10% of the study participants in the qualitative sub-study, to provide a wide range of experiences and views. Data collection took place concurrently with recruitment, preventing calculation of percentage recruitment. Hence, interviews were carried out with as many people as possible on data collection days in each region.

Interviews included 27 people who completed the intervention; 20 people who dropped out of the intervention; and 18 people in the usual care arm of the study (16%, 40% and 13% of total trial participants, respectively).

Twenty-six participants were women (46%) and 31 were men (54%), and they varied by age (mean 60 years), and duration of dialysis (mean 43 months). They all received hemodialysis thrice weekly for 3.5–5 h. On average, interviews took place 11 months after informed consent was received. All trial participants who had received an invitation letter and who were available for interview on the qualitative data collection days agreed to participate. This resulted in 895 min of interview (mean of 14 per person, range 4–35 min), 133,405 words of interview text (495 pages of transcription).

In total, 39 “providers” were also interviewed, including five Renal Consultants, nine PEDAL employees who provided the intervention to participants or were involved in managing the trial, six Nurse Managers/Advanced Practitioners in the Renal Unit, nine Nurses and ten Health Care Assistants. Some providers were unavailable on data collection days but no one refused to participate for other reasons. This resulted in 739 min of interview (mean of 18 per person, range 6–53 min), 120,826 words of interview text (349 pages of transcription).

**Figure 1:**
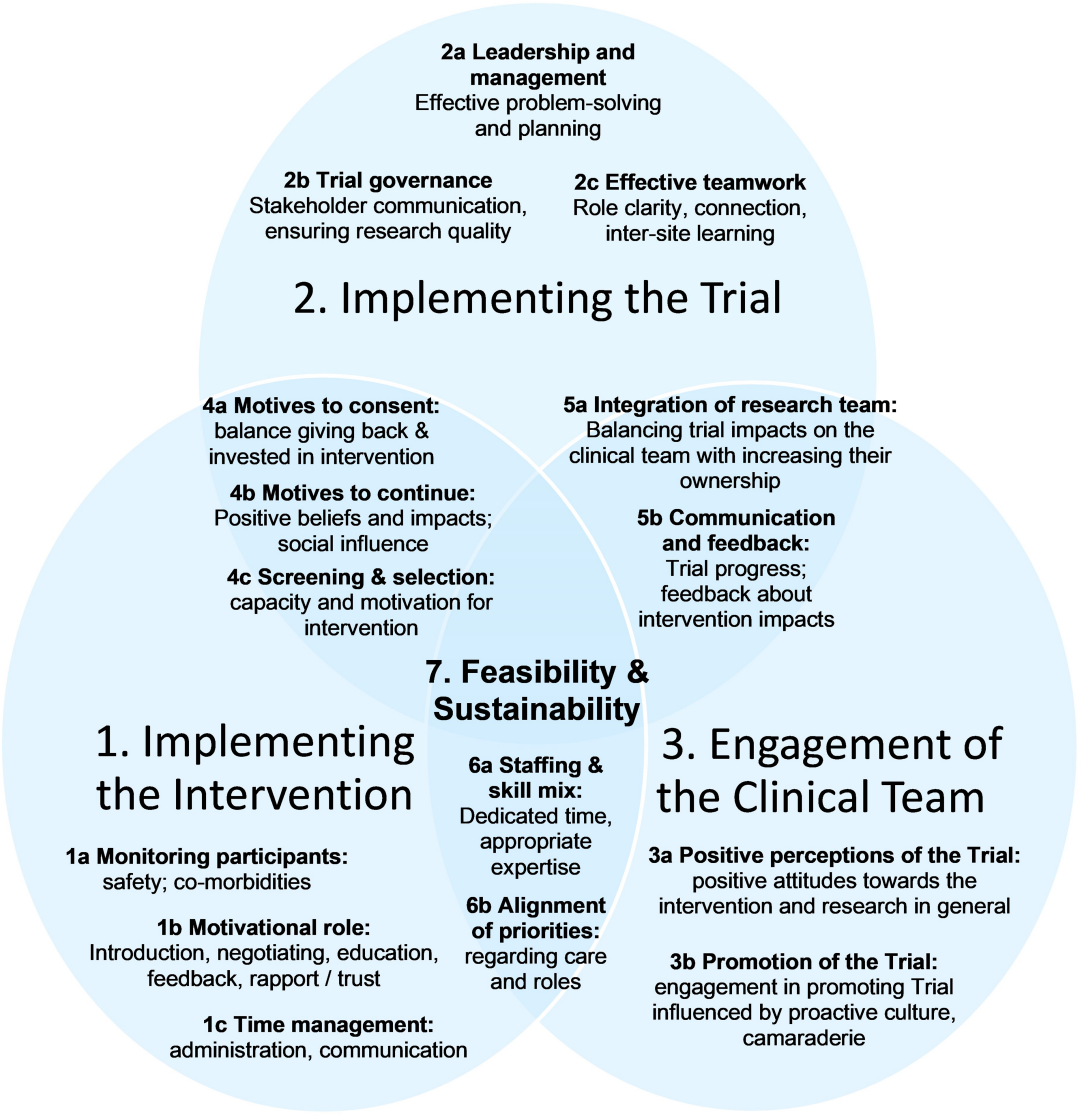
Explanatory theory: the ideal scenario.

In-depth thematic analysis led to the development of explanatory theory, named “the Ideal Scenario,” illustrated in Figure 1. It reflects complex interactions between different aspects of intervention delivery and trial implementation with the clinical environment. Aspects that were optimal in different sites are represented, based on descriptions of what was and was not conducive to sustainable trial delivery and longer-term intervention sustainability. Three “initial themes” emerged: (1) Implementing the Intervention; (2) Implementing the trial; and (3) Engagement of the clinical team. Each theme is explained, followed by Table 1 which summarizes sub-themes, their definitions, and illustrative quotes (labelled to enable cross-referencing within the text). Interactions between initial themes 1–3 are explained next as “linking themes” 4–6, with evidence provided in Table 2. Finally, Theme 7 explains the “big picture” of feasibility of the trial and sustainability of the intervention in the long-term (evidence in Table 3). Quotations are drawn from the Provider interviews due to the focus of this article, and these are consistent with evidence from Participant interviews (19).

**Table 1:** Summary of overarching themes and definitions with example data.

**Sub-theme names** (grouped by Theme – named in dividing rows)	**Definitions** (descriptions of each sub-theme to increase auditability in relation to the “fit” of quotations with each sub-theme)	**Illustrative quotations** (Quotation references e.g. 1ai preceding each quotation enable the reader to locate the correct quotation relating to the results in the text, providing evidence of each point in the text)
**Theme 1: Implementing the intervention**		
1a Monitoring participants	Comments describing the importance of monitoring patients for: decreased blood pressure, fistula management, falls, temperature, blood sugar levels, and heart rate; being prepared for patient condition to change quickly.	Quote 1ai: It's getting them familiar with the bike and then teaching them the muscle conditioning exercises and just checking their blood pressure throughout, obviously feed back to the nurse if there's any problems or if the machine's going off perhaps. …so I’ll have a chat with them whilst they’re cycling and want them to feed back on how they’re feeling. [Provider 1: London]
1b Motivational role	Comments describing important roles of the people implementing the intervention, including: introducing the trial to potential participants; using education to persuade people to participate; negotiating and convincing participants to engage in the intervention on a regular basis; using feedback to encourage; providing positive company; developing rapport and trust.	Quote 1bi: I talk to them about the benefits of cycling and I remind them of how they felt last time, because I find generally patients after they’ve cycled do feel good and they enjoyed it and they’re feeling happier with themselves, and then it's kind of negotiating, well, how about we do five minutes less. So I do try to be as encouraging as I can but not pushy, because I know that when they’re feeling rubbish … I mean, when I’m feeling ill the last thing I want to do is cycle, so I can understand, so showing a bit of empathy. [Provider 1: London] Quote 1bii: … quite often you’re motivating them. Because you’ll come in and they’ll be like, oh I didn’t sleep very well, I can’t be bothered doing this today … just to encourage them. It's part of my roles I suppose, encourage them more and keep them going. …And we can quite often have a laugh with them while we’re doing it, so we’ve quite bonded. I think they look more forward to the banter than the bike, but some of them really enjoy it. [Provider 1: Central Scotland]
1c Time management	Comments relating to the importance of managing one's time effectively to optimize intervention delivery, including recruitment, communication, organization, and optimizing the numbers of participants seen each day.	Quote 1ci: I consented the new patients, … it's obviously booking them in for their baseline assessment with the research doctor, reminding them of their appointment, booking them transport … [Provider 1: London]
**Theme 2: Implementing the Trial**		
2a Leadership and management	Comments that expand on the need for leadership and management in implementing the Pedal Trial, including the need for ongoing problem-solving and planning relating to: complexity of funding arrangements and trial set-up; adjustments to the equipment; support staff recruitment and time management; and challenges with participant recruitment.	2ai: … there are a few things that I hadn’t realized. One is the setup, it's quite complicated and takes time, takes six months to set up the whole thing. There are certain elements that are not funded and getting funding for that was quite difficult, quite a struggle. We managed in the end. So, for example, we did not have a CPET machine for assessment of exercise, which you had to buy … [Provider 2: West Midlands] 2aii: The most recent example is the employment of the physiotherapy assistants … the money for the physiotherapy assistant was actually classified as excess NHS costs … so we had to apply separately, locally … So that was a huge delay at the start because it means that immediately you don’t have everything in place to actually just start organizing everything so that delayed things. [Provider 6: Central Scotland]
2b Trial governance	Comments relating to the importance of ensuring that the trial is conducted according to the protocol, involving communication between different stakeholders and challenges in balancing different priorities.	2bi: It's been good, not perfect. There's been a lot of teething problems, maybe some misunderstandings between the University side and the physiotherapy side, obviously, expectations to reality. [Provider 1: Central Scotland] 2bii: I just think over time if you’re busy you forget, that's probably another reason, and I know I said, has the patient been offered it? And he's like, well, no, I was really busy. Or I was asking why the book hadn’t been written up and he said, oh, he was really busy and he just never wrote up the book … I think sometimes it does get forgotten and part of that is being busy as well and short-staffed. [Provider 3: Central Scotland]
2c Effective team work	Comments relating to the value of ensuring effective teamwork, including meetings to ensure people feel connected and have clarity about their roles and responsibilities.	2ci: So when I first started obviously [NAME] has been here from the beginning, so she knew a lot about the study and it was only really when she left that she handed over stuff to me … but there's all the things I didn’t realize she actually did. And a couple of things that hadn’t gone as well in the study as we’d hoped, so it was things like bloods not being done … so I was responsible for chasing them up, I was also responsible for booking the patients’ transport to and from their assessments. So then I also needed to book patients in for their assessments which I wasn’t doing previously. I’m trying to think what else I do. Then there's things like the e-CRF, which is something that's new and we need to input the data … it would also help to have more regular team meetings. [Provider 1: London] 2cii: Yes. The two managers have been … very encouraging. They’re always there if you need them. [Provider 1: Central Scotland] 2ciii: Because, you know, we feel now that we’re working in isolation, so we’re just saying, I wonder what's happening at other sites? I wonder if other people are experiencing this? … But the actual team, you know, there's no information coming now that we can share, … in terms of what's happening at site it's felt quite hands off. [Provider 8: East Midlands]
**Theme 3: Engagement of the Clinical Team**		
3a Positive perceptions of the trial	Comments demonstrating the need for positive perceptions of the intervention and its trial context among people in the clinical team, including: attitudes towards the intervention balanced with negative aspects of the trial (e.g. working with disappointment when people are allocated to the control group; attitudes towards research	Quote 3ai: I actually think very positive of it. Yeah, I think it's a great idea, ‘cause patients spend half their life on the unit. And if they can make them physically fitter, I think it's great. All for it. [Provider 4: West Midlands] Quote 3aii: Positive, yeah very positive. I’m biased though so. I think it's going to work. I mean the evidence that is out there in different populations is quite convincing, so. I think it's a shame we haven’t done it for so many years. [Provider 2: North Wales and North-West] Quote 3aiii: I think, yeah. Some of the patients that are on the study are a lot more positive. They’ve come in a lot more vibrant; they’ve come in happier, you know, their mobility's better so that we’re not using wheelchairs or transferring as much. So, yeah, I do think it has made our life easier a little bit as well. Even if it's just, you know, a small bit it does make a difference. [Provider 6: East Midlands] Quote 3aiv: They’re good, they do research here all the time. So the staff are very aware of research going on, and they are very accommodating with, you know, they’ve looked after us really well. And they do try and encourage the patients … [Provider 1: West Midlands]
3b Promotion of the Trial	Comments relating to promotion of the trial, influenced by the clinical team's culture (e.g. proactive; generating camaraderie in the Renal Unit)	Quote 3bi: … we like embracing new things on the unit. … I don’t really think we have a problem with change on this unit, I think we embrace it. It's new at first but you adjust to it, no trouble. We seem to do that here. So, with the bikes, we were looking forward to getting the bikes and getting them on board because we wanted to do that for a while, for a few years now. So, we were glad about it really, yes. [Provider 5: North Wales and North-West] Quote 3bii: we’ve done things down here that we’ve tried to motivate the patients with. … staff are quite motivated … I mean, that's an enormous factor when you’re trying to recruit for something like this. Staff are motivated. [Provider 2: Central Scotland] Quote 3biii: I think probably what happens in the renal units … they’re not very enthusiastic or proactive about implementing an exercise programme. I think it's because it's not considered to be high up in the priority list because we have units … that are very enthusiastic, there's the staff, the nurses, the support workers are very happy to actually take that role and push the exercise idea with the patients. They do that as part of their job even though it's not clearly defined in their job description. Then you have other units that we tried to bring into the trial and they’re just not even responding, to emails and to even just arrange a meeting. [Provider 6: Central Scotland]

**Table 2:** Summary of overlap between initial themes with definitions and example data.

**Sub-theme names** (grouped by Theme – named in dividing rows)	**Definitions** (descriptions of each sub-theme to increase auditability in relation to the “fit” of quotations with each sub-theme)	**Illustrative quotations** (Quotation references e.g. 1ai preceding each quotation enable the reader to locate the correct quotation relating to the results in the text, providing evidence of each point in the text)
**LINKING THEME 4: Implementing the intervention interlinkages with Implementing the Trial**		
4a Motives to consent	Comments that illustrate different reasons for people agreeing to take part in the study, feelings about data collection on their non-dialysis days, and the influence that these issues can have on their attitude toward randomization and ongoing trial participation.	Quote 4ai: And it's a shame because you’re not getting your pedallers. You’re just getting those people that have kind of being talked into it, no, I don’t really want to ride, and they’re the ones that get randomized to ride. [Provider 9: East Midlands] Quote 4aii: … one of them was bitterly disappointed to be randomized to control so much so that when it came to his follow up he absolutely…I’m not interested, you know. [Provider 8: East Midlands] Quote 4aiii: On paper it looked good, and it looked like it would work. And then when we started setting it up … it was looking like it would be more tricky to actually recruit. It seemed easy on paper, but when you start looking through it, and looking at your patients you realize there's going to be obstacles that we were going to get stuck with. …obviously the patients come here three times a week, and the assessments are a non-dialysis day. And when you speak to the patients their days are precious to them, so they don’t like interrupting their two days off to come into hospital for two hours. [Provider 1: West Midlands]
4b Motives to continue in the Trial	Comments that explained why people continued to participate in the intervention or control group over time or did not continue. These were influenced beliefs about the intervention, social influences, and positive experiences of the intervention.	Quote 4bi: The other funny thing is, the people that were really keen to go on the exercise were allocated randomly to non-exercise groups, so we ended up with a group of people in the non-exercise group that were enthusiastic about exercising, and the exercise group people that were not that keen, they just agreed to take part. As a result, we had a few drop-outs … [Provider 2: West Midlands] Quote 4bii: It's unfortunate the randomization … it disappoints … somebody's just been turned down and you’re exercising somebody in the next bed to them. How do you keep them interested in the trial for nine months now? [Provider 4: North Wales and North-West] Quote 4biii: I’ve got a couple of people that spring to mind that refuse to do it [intervention] pretty much every time I see them…. But you have some people I feel that stating the benefits encourages them… [Provider 1: London] Quote 4biv: seeing the difference in a handful of patients, you know, with their walking, with their mobility, with their mood, you know, doing something to side-track them from dialysis, but also seeing the strength gained in their muscles, in their legs, in their mobility - so it's changing their whole outlook on their time here. [Provider 6: East Midlands]
4c Screening and selection of participants	Comments relating to potential participants’ and actual participants’ capacity for intervention, including existing or newly developed co-morbidities and lack of motivation for the intervention.	Quote 4ci: A lot of my patients are part of it … it's more making sure that they’re fit enough to be going near and exercise programme … Because a lot of patients are frail and have cardiovascular disease and things, so can’t. [Provider 6: London] Quote 4cii: Or even just making sure that the ones that you’ve put on it… wouldn’t be patients with hip and heart conditions … I mean people would be more suitable for doing it, and be motivated to do it. Maybe not. They just have to not have problems that some of the patients we’ve had that have had to drop out, if you know what I mean? [Provider 1: Central Scotland]
**LINKING THEME 5: Implementing the Trial interlinkages with Engagement of the Clinical Team**		
5a Integration of research team within the Clinical Context	Comments relating to the way in which the trial was implemented and its impacts on the Clinical Team	Quote 5ai: Initially, when they first turned up, I might have spent an hour, where are they going to go, but after that it's been absolutely no trouble for me whatsoever. [Provider 3: East Midlands] Quote 5aii: … doing all the paperwork, the preparation work in the bike set up… As I say, it is a busy unit. There was some concern … before we realized that we wouldn’t have to do it ourselves, there was concern that … whether we’d have time and how it will impact on our … on the shifts … And that continued I think right up to when we realized we wouldn’t have to do anything. [Provider 5: East Midlands]
5b Communication and feedback	Comments relating to communication and feedback between the trial team and the clinical team, including the importance of communication to influence understanding and perceptions of the trial and its progress and feedback evident from intervention participants about its positive impacts	Quote 5bi: … to sort of quantitatively assess effectiveness and this and that is all very well and good, but what that means to our patients is more important for us as nurses here, how that affects our patients in the way they feel about themselves is far more important to us than any statistical representation of study has meant for someone else. [Provider 5: Central Scotland] Quote 5bii: I think for the patients, seeing the difference in a handful of patients, you know, with their walking, with their mobility, with their mood, you know, doing something to sidetrack them from dialysis, but also seeing the strength gained in their muscles, in their legs, in their mobility so it's changing their whole outlook on their time here. [Provider 6: East Midlands] Quote 5biii: if I’m honest I thought, where the hell are we going to keep these bikes? … But seeing the difference that it's made to the patients I think it's a marvelous thing, I really do. I did worry how we were going to get it under the chair. Is it going to affect the needles? Is it going to affect the access? Is it going to make the machines alarm? But, no, we’ve had none of that, so my worries were eased quite easily. [Provider 6: East Midlands] Quote 5biv: I definitely think the trial and the intervention is, from what I’ve seen, it certainly works. Although I’ve only seen a small snippet of the evidence, it certainly seems as if it does work. It looks as if it is helping with clearances, it's making the patients physically stronger and healthier. It's helping their blood pressures, heart rates, it's helping with the patients’ confidence. [Provider 1: Central Scotland]
**LINKING THEME 6: Engagement of the Clinical Team interlinkages with Implementing the Intervention**		
6a Staffing considerations	Reasons that providers gave for the importance of dedicated staff time and appropriate expertise during the trial).	Quote 6ai: I think the different strategy would need to be … I think, you know, depending on how successful they wanted this PEDAL Trial to be, … I think if it was to be successful, if there was something successful coming from it, then maybe, you know, maybe we would have … to say we really need a person, one person to be able to detach themselves from other things that are happening in order to oversee all this. [Provider 2: Central Scotland] Quote 6aii: Well the thing is, because we’re always short staffed, I hope in future if the PEDAL roll out is like a must during dialysis … I hope they won’t pinch any of the nurses from our nursing duty to do that. Not that we’re not willing. But the thing is, I think priority is dialysis first and there are things that, we as nurses, we’re not as good in the way like the proper physio is. Like for things like if they got like a prescription for them and it's all tailored made for them. … But for us we only do the observations and the vital signs, that's our nursing bit. [Provider 6: North Wales and North-West]
6b Alignment of priorities	Culture, leadership, Themes relate to staff interest in the study and variation in units Comments relate to various reasons why staff were/were not interested in the trial. Staff were interested in research, improving career prospects, and felt the study asked a valid research question. Staff were not interested because: they were too busy, short-staffed, and felt it was not a priority for the unit	Quote 6bi: Oh without a doubt, without a doubt. If it can be proved that it helps the patients, then I think without a doubt it should be integral to the role. … Well, the fact that I can see physical benefits made in the patients then, to be honest if it's helping the patients I’d happily do anything, to help them, you know what I mean. That's what I’m here for, that's what I’m paid for, you know what I mean. In my opinion it doesn’t matter if you’re rushed off your feet, you’re paid to be here so you’re paid to be rushed off your feet. [Provider 1: Central Scotland] Quote 6bii: To sort of quantitatively assess effectiveness … is all very well and good, but what that means to our patients is more important for us as nurses, how that affects our patients in the way they feel about themselves is far more important to us than any statistical representation of the study: [Provider 5: Central Scotland]

**Table 3:** Summary of data supporting interlinkages between all overarching themes in relation to trial feasibility and intervention sustainability.

**Sub-theme names** (grouped by Theme – named in dividing rows)	**Definitions** (descriptions of each sub-theme to increase auditability in relation to the “fit” of quotations with each sub-theme)	**Illustrative quotations** (Quotation references e.g. 1ai preceding each quotation enable the reader to locate the correct quotation relating to the results in the text, providing evidence of each point in the text)
**OVERARCHING THEME 7: Trial Feasibility and Intervention Sustainability**		
7a Feasibility of the Trial:	Complex interactions across themes, exemplified by more detailed descriptions from Providers which explore: • the trial structure (randomization to a control group, burden of assessment, inflexibility of the intervention regimen) leading to negative experiences for those who could/did take part and for those recruiting to it • cultural context of trial implementation as influenced by leadership, camaraderie, providers’ attitudes towards their roles • impacts on success of the trial from the person who is “on the ground” communicating with everyone involved	Quote 7ai: I don’t understand why if you’ve got to do assessments on patients just do it before. Have a room where you can have whatever and … do it there. I think the randomization of the study as well doesn’t help because you’re having people that really do want to pedal and maybe are randomized into not pedal which is not cool. [Provider 9: East Midlands] Quote 7aii: Well, as I said about the patients, if they’re not motivated to do it, it can actually end up being de-motivating for staff - because they’re asking, do you want to do this? And they’re saying, no, I don’t want to tonight, I can’t be bothered tonight or whatever. It ends up being de-motivating for staff and then they stop asking over time. … Ah huh, and it's quite hard to get that motivation back, so there's that. And I know I find that from my point of view you try and introduce stuff to patients and there's a lack of interest or you get negative comments or whatever and it does de-motivate you. … And I think possibly the nurses as well have been disappointed … because there's been certain patients that we felt - he would really benefit from being on that bike, and he wasn’t randomized to be on the bike. So that's disappointed a lot of people and I don’t know if that then de-motivated people as well because the study's not doing what the nurses think it should be doing, which is your younger, fitter patients that could really benefit from it. [Provider 3: Central Scotland] Quote 7aiii: now I think we are wiser. So, if you do a study in the future, I think we’ll do all this. We’ll give the patients a bit more freedom, we’ll give the nurses a bit more freedom, and make sure that we are not so strict with the exercise regime because, the stricter you are, the less likely you get people to buy-in. [Provider 2: West Midlands]
7b Sustainability of the intervention in the long-term	Complex interactions across themes, exemplified by more detailed descriptions from Providers which explore: • experiences of the trial alerting people to potential for changes in their roles and resistance to such changes • practical implications of implementation and conflicting demands on people and space • contrasting attitudes, approaches and barriers in relation to empowering and enabling people • embedding the intervention within the service consistently with growth of trust and relationships	Quote 7bi: It's difficult to sort of have a feeling about it. It's difficult to have any opinion on something that you know from speaking to patients that it's had quite a positive contribution in many of their lives. You feel like you’re quite glad it's going on, but at the same time you’re also quite glad that it's not adding to your workload as well. It's helpful that it's someone else's job to do it. [Provider 5: Central Scotland] Quote 7bii: Of course there are other units that are not really built to facilitate any intervention like this or they don’t have space, enough space for storage, for example, or if you actually go and put a bike in front of the patient's chair it may compromise health and safety because there is just enough space around the chair in case anything goes wrong to access the patient and do what they have to do, you know, to resuscitate the patient or whatever. So there are a lot of issues, I guess, to be resolved. I think it all goes back into if you are to do something like this and develop this as a service you have to go to the very start and actually build it in the jobs in the unit from the very beginning where you have everybody involved. They know it's there to be delivered and it's not like an *ad hoc*, something that is added on as an afterthought because it's just not going to work. You have to have everything in place for this to work. The space; the storage; the staff; the money, the funding; everything, otherwise it's just not going to happen. [Provider 6: Central Scotland] Quote 7biii: I mean, I’ve said to [Trial employee], what I’d love to see, come the end of this study, is because we get to keep the equipment, is actually have one of our bays sort of turned into a bit of a gym … so patients that are like minded … can have the bikes and maybe even have some weights and thing … Certainly when the bikes came in we had a bit of fun with them ourselves, getting the staff to pedal with them and try different things just to get staff interested. [Provider 9: East Midlands] Quote 7biv: I think if staff have a little bit more involvement it will make them more proactive and maybe it will make them ask that question to the next patient. … I would love to see a lot more exercise on the unit. I want to see my patients feel better and have better outcomes. [Provider 9: East Midlands] Quote 7bv: … it should be that culture change happens almost by default because we think, come on, this is good for you. You know, just walk. Just walk to the scales. I’ve brought you in, but I want you to walk to the scales rather than me wheel you to the scales. That little bit of, because I know it's good for you. [Provider 1: North Wales and North-West] Quote 7bvi: I think it's multifactorial. I’ve thought a lot about this … Of course, you can come in and do it yourself and the biggest resistance I had was not the patients but the nurses and a lot of it came from … it's a bit like your children, “oh, I’ll do it myself, so it’ll get done properly.” It takes too long when they do it, they don’t do it properly; so you have that maternal or paternalistic approach of, I’ll do it and I know it's done well or it's done properly or I can get it done quicker. … they’re doing it because they care and don’t want them to get worse…. [Provider 1: North Wales and North-West] Quote 7bvii: … in the past I’ve run trials in CKD patients and dialysis patients that look at the effects of exercise vs. control group … I was responsible for doing everything … from patient recruitment to assessment to training. That gave me the opportunity to develop a better relationship with patients. The patients got to know me. I think in the end you need to have that level of trust between whoever works with patients and an external person for these kind of interventions to work. I think that's what made the whole difference in the past … when I was running exercise classes for dialysis and sessions of dialysis for CKD patients, people actually kept coming even though … they were not part of the trial anymore, just because they liked the experience and just because they knew there was a service and facility there. They had participated, nothing bad had happened to them so they kept coming just because they knew me, and the service was there. [Provider 6: Central Scotland]

### 3.2. Theme 1: implementing the intervention

Within Theme 1 pragmatic tasks required for the intervention are described, such as monitoring people and ensuring their safety (Quote 1ai), and more in-depth communication with people on each attendance day to encourage and motivate them (Quote 1bi, 1bii). The behavior change element of the intervention was not easy for people with variable health status and support for this required a careful balance of encouragement and persuasion with respect for the person's decision. Managing this alongside administrative tasks required well-developed time management skills (Quote 1ci).

### 3.3. Theme 2: implementing the trial

Implementation of the trial was demanding (Theme 2). Strong leadership and management were necessary to enlist different sites in the trial, work with the complexities of funding in different sites, ensure that equipment was appropriate and in the right places, recruit and train staff, and manage the ongoing trial requirements (Quotes 2ai, 2aii). Leadership was crucial to trial governance to ensure effective communication between all stakeholders (Quote 2bi), optimize the pace of participant recruitment, and ensure that protocols were followed despite other clinical demands (Quote 2bii). This required effective teamwork within each site in relation to role clarity and support when needed (Quote 2ci) and between sites to ensure that people felt connected to the wider trial team and could share strategies (Quote 2cii).

### 3.4. Theme 3: engagement of the clinical team

Successful recruitment of participants and their ongoing participation in the trial was influenced by engagement of the clinical team (Theme 3). It was important that providers had positive perceptions of the trial and the intervention. Some providers were supportive of the intervention due to consistency of its focus on exercise with their personal beliefs (Quotes 3ai, 3aii). Some had negative expectations but were pleased that the trial had fewer impacts on their workload than expected. Others were encouraged when they saw positive impacts of the intervention on participants (Quote 3aiii). Engagement in promoting the trial and intervention was higher where providers had a positive attitude towards involvement in research and development (Quote 3aiv), which was influenced by the culture within the clinical environment. For example, participants in some sites described embracing change, motivated teams, and willingness to engage with new aspects of their role. They also explained how this impacted on their actions, such as being very proactive in talking to possible participants (Quotes 3bi–iii).

### 3.5. Linking theme 4: implementing the intervention overlapping with implementing the trial

Linking Theme 4 demonstrates the complex balance between maintaining trial fidelity and ensuring trial recruitment. The burden of data collection for the participant was a barrier; people were required to attend for this on a non-dialysis day, potentially at a substantial distance from their home (Quote 4aiii). Some people agreed to participate because they were highly motivated to participate in the intervention, while others were motivated to “give back” to the service (Quotes 4ai–ii). These contrasting motivations influenced ongoing participation in the trial. If people who wanted to exercise were not randomized to the intervention group it was much harder to encourage ongoing participation in the usual care group, with data collection sessions on days of respite from hemodialysis (Quote 4aiii, 4bi–ii). In contrast, people with less interest in exercising found it difficult to sustain participation in the intervention group (Quote 4biii), although this was sometimes positively influenced by the benefits of taking part (Quote 4biv). Some people were more suitable for the intervention than others, with people having to stop participating early due to developing comorbidities (Quote 4ci). Providers also commented about characteristics that they felt were likely to affect success of the participant in the trial, such as low motivation (Quote 4cii).

### 3.6. Linking theme 5: implementing the trial interlinkages with engagement of the clinical team

Linking Theme 5 describes how trial feasibility was influenced by clinical team engagement. Minimal impact of trial delivery was seen as important and there were multiple descriptions of providers being pleasantly surprised that this was the case (Quotes 5ai–ii). There was a risk that this led to lack of engagement of the clinical team with the trial who did not then develop a sense of ownership. It was important that the trial team were communicating about how it was progressing, including what stage they had reached in relation to timescales. Clinical team members also found it very motivating and engaging when they saw people responding well to the intervention (Quotes 5bi–iv).

### 3.7. Linking theme 6: engagement of the clinical team interlinkages with implementing the intervention

When considering potential long-term implementation of the intervention, the clinical team had specific thoughts relating to the amount of staff time and specific expertise required (Quote 6ai–ii). Views seemed to be affected by the degree of alignment described between the clinical team's priorities and those of the research team. Some clinical team members described being willing to make changes to their role if it would support the wellbeing of service users (Quote 6bi). One person explained that the value of the intervention must be clear from the changes they see in their service users (Quote 6bii).

### 3.8. Overarching theme 7: trial feasibility and intervention sustainability

The results of detailed analysis suggest enormous complexity in running a trial successfully in multiple contexts. An RCT structure has elements that may be difficult to accept for people who are delivering a service and provide support and care to individuals over a sustained period. It is painful to see a person allocated to the control group when they joined the trial with a strong desire to participate in the intervention (Quote 7ai–ii). It is hard to persuade people who are suffering to give up precious time away from the hospital to do assessments. It is demotivating to be criticized for not recruiting enough people or implementing the intervention exactly as it is written on paper (Quote 7aii–iii). Analysis suggests the greatest likelihood of a trial being successfully delivered comes from a supportive culture in the clinical environment and highly effective communication between the trial and clinical teams. Even where there is a highly proactive culture in the clinical environment there must be clear explanation of reasons for decisions, flexibility wherever possible, and ways of compensating those in the control group for the loss of the intervention experience.

Ensuring that a trial does not impact heavily on the clinical environment is intuitively appealing. It is more likely to be accepted within the clinical environment and achieve greater standardization across different sites. This prioritization of trial integrity may have a detrimental effect in the long-term, however. Firstly, even with minimal impact on workload, implementation of the trial is highly visible to people working within the clinical environment. They have time to think about what it could mean for them in the long-term, while not necessarily feeling invested in the outcome. In some sites, this led to reservations or resistance in relation to long-term implementation without substantially increased resources (Quotes 7bi–ii). In trial sites with less reliance on people employed through the trial, involvement was driven by a proactive culture and belief in a wider role of the provider (Quotes 7biii–vi). One site aimed to develop camaraderie within the clinical environment and provide different activities to engage people during Hemodialysis. Due to less resourcing of intervention delivery, it was not as “pure” as at other sites, due to competing demands of staff. It was noticeable that people spoke more positively about implementation in the longer-term without this requiring external resourcing, for example, by integrating a new role into future job descriptions. At another site a trial employee who was already embedded in the wider team invested substantially in communicating with everyone in the clinical environment. One interview with the person who stored the equipment illustrated initial frustration with the increased challenge to available space, which was ultimately converted to enthusiasm about the intervention and involvement in camaraderie with participants. Such strategies embed the intervention over time, increasing the likelihood of longer-term implementation. Another interview illustrated that such embedding may also increase the likelihood of participants continuing to engage with the intervention beyond the trial, due to feeling safe and trusting the providers (Quote 7bvii).

## 4. Discussion

This qualitative analysis addressed questions relating to feasibility of the trial and sustainability of the intervention in the long-term and how insights can be used to inform future rehabilitation intervention evaluation studies with a view to implementation. Three key ideas will be discussed in this section, informed by the research findings. First, “The ideal scenario” explanatory theory aims to provide insights for people who are planning RCTs with contextual similarities to the PEDAL Trial. This could be used to support pre-emptive and ongoing analysis of risks to future trials and feasibility of their delivery over time.

Second, the possibility is discussed that other types of study design may suit many rehabilitation contexts better and lead to more informative results. Quantitatively, the PEDAL Trial fell short of statistical significance (*p* = 0.055) for the intervention, while qualitative evidence suggested that it could have substantial positive impacts for some people (see Table 13 and pages 34–39 of the original project report (19). Other types of study design might analyze this more usefully and provide more valuable conclusions for policymakers.

Third, some study findings suggested that the trial context might lead to negative perceptions of the intervention within the clinical setting that may jeopardize longer-term implementation. The potential for integrating implementation research earlier in the research to practice journey is discussed further.

### 4.1. Insights for future RCTs

RCT design and delivery requires a complex interplay between individuals with different roles, operating within different systems, and with different agendas. The person receiving the intervention is at the center of this, as it is their decision to engage with the trial and intervention. Many things can influence this decision, however, including the person's beliefs and priorities, the culture of the clinical environment, and strategies used by the trial team.

To facilitate consideration of whether “The Ideal Scenario” explanatory theory may be useful to your context, we have identified some key characteristics of the PEDAL Trial context. These involve:

• implementation of the trial within different sites, each with an integrated community of providers and potential participants which differ in leadership and culture;
• the possibility that ensuring trial and intervention fidelity may conflict with ability to recruit and maintain participation;
• evaluation within a social context of an intervention that involves a complex behavior of varying appeal to participants; and
• evaluation of an intervention which may be viewed as beyond the current scope or roles of key clinical providers.

For trial planners who see commonalities between their context and that of the PEDAL Trial, it may be useful to use the explanatory theory within a risk assessment process. Each aspect of the theory represented in Figure 1 can be considered carefully during the planning stage of a trial and then later as the trial progresses. Where a site identifies risks, problem solving can be used where possible to mitigate these and reduce their impact on trial recruitment, retention, quality, and likelihood of implementation post-Trial.

### 4.2. Insights suggesting alternative study designs focusing on impacts of an intervention

In some circumstances alternative study designs may be more informative and potentially more philosophically aligned with the worldwide aspiration to person-centered practice (21, 22).

Our results demonstrated challenges in recruiting and retaining involvement of appropriate clinical sites and participants. The burden of data collection influenced both consent and completion of the trial, as did randomization of participants to intervention and control groups. When designing an RCT optimizing external validity often requires inclusion of as many people as possible, minimizing exclusion criteria. This can mean that people who are less likely to benefit from the intervention are included because there is no specific reason that they should not participate. A contributing factor to the PEDAL Trial's lack of significant improvement in quality of life was the low compliance rate of 47% of sessions completed by participants, and an even lower percentage of engagement (18%) in the prescribed exercise intensity and duration. For many participants the training load and duration were insufficient to elicit physiological changes with potential to impact upon health-related quality of life. Qualitative findings provided insights into the barriers to participation (19). These challenges ultimately influenced the final quantitative results being unsupportive of the intervention effect despite highly supportive qualitative findings for some participants.

Bonnell and colleagues (23) argue for conducting Realist RCTs when evaluating complex public health interventions. They contend that RCTs generally fail to explore the ways in which aspects of the intervention and the local evaluation context interact. In contrast, Realist evaluations develop prior theory about what works, for whom, and in what circumstances. The Realist paradigm sees evaluation as exploring “an open system of dynamic structures, mechanisms and contexts that intricately influence the change phenomena that evaluations aim to capture” (23 *p*. 2,299). Bonnell et al. (23) argue that neither RCTs nor Realist approaches achieve everything that is needed and merging the two is optimal. The RCT enables testing of a potentially causal mechanism under optimal conditions; meanwhile, further contexts can be investigated in relation to influences of unexpected underlying mechanisms on outcomes in a Realist Evaluation. They argue that most interventions will impact positively on some people, in some conditions, which is the most important information for policymakers.

While the PEDAL Trial took place in clinical settings, rather than a public health context, the needs of the evaluation appear to be similar. Public health interventions are described as complex social interventions, in contrast to pharmacological ones, for example, and interact with their contexts in different ways (23). Our qualitative analysis suggests that that there were also numerous social influences on the PEDAL Trial. Bonnell and colleagues argue that most RCTs collect information that would enable development of mid-level programme theory (23). To explore this suggestion, some of the qualitative results of the PEDAL Trial reported by Greenwood et al. (19) have been reformulated into a “Context-Mechanism-Outcome” (CMO) statement: “People who had sufficient motivation for the intervention and good enough health status (Context) and who were in a clinical environment that cultivated positive and empowering relationships (Context and Mechanism), were more likely to continue exercising while undergoing hemodialysis three days a week (Mechanism) in Renal Units which promoted the Trial (Context and Mechanism), leading to improvements in their physical, functional, psychological and social wellbeing (Outcome).” When constructing this statement from key study findings we found overlap in what can be considered context and mechanism. It would be interesting to explore the validity of this statement using post-hoc quantitative analysis.

Bonell et al. suggest placing greater emphasis on ensuring trial fidelity in relation to the processes and functions within the intervention (mechanisms of change), rather than precise activities (23). Ongoing qualitative research could explore ways in which mechanisms and outcomes differ at each trial site and quantitative data can be used to test evolving theories. In this way, a Realist RCT can explore validity of the theory as well as effectiveness of the intervention. If supportive, this theory could then be applied more flexibly in different clinical contexts, supporting long-term implementation.

### 4.3. Insights suggesting alternative study designs that consider long-term implementation

PEDAL Trial analysis found challenges in reconciling the competing demands of reducing impacts of the trial on the clinical environment in the short term, and enlisting providers to enable implementation in the long-term. Funding of trial employees may optimize trial fidelity; however, local providers are less likely to have a sense of ownership of the intervention. Local providers can also become alert and resistant to possible future changes in their role. By collecting qualitative data from sites in all five study regions it was possible to compare different scenarios within the PEDAL Trial. There were clear reservations about intervention implementation in sites that were experiencing greater pressures relating to staffing and morale, were less proactive about research, and where people had a less empowering and more maintenance-driven approach to their role. In contrast, some sites had proactive, enthusiastic cultures, interest in research, and desire to support camaraderie, independence, and wellbeing in their community of service users. In the latter, there was greater emphasis on problem-solving when discussing longer-term implementation.

**Figure 2:**
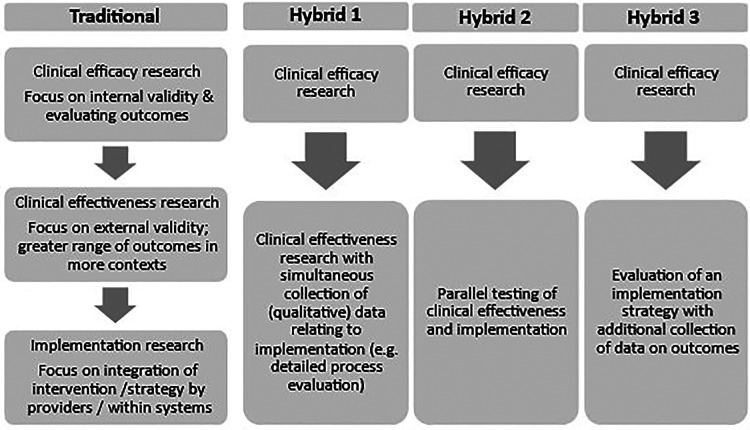
Summary of a traditional model of progression from clinical efficacy to clinical effectiveness and implementation research and three proposed effectiveness-implementation hybrid designs [based on information from Curran et al (1)].

It is possible that where there is existing evidence to support an intervention, an alternative study design would enable a more positive change management process that does not risk alienating providers. Glasgow et al. (24) argue that RCTs focus on internal validity at the expense of important questions relating to how the intervention might be implemented in varied contexts and maintained over time. Curran and colleagues (1) advocate for “effectiveness-implementation hybrid designs” in some circumstances, to blend clinical effectiveness and implementation studies and thereby reduce the time lag for knowledge translation and develop more insightful implementation strategies. Figure 2 compares the traditional research journey with three different suggested hybrid approaches (1) which aim to integrate exploration of clinical uptake of the intervention with effectiveness evaluation.

Considering Figure 2, the PEDAL Trial followed a primarily traditional pattern and was situated within “clinical effectiveness research”, building on earlier efficacy studies. The qualitative sub-study collected detailed data to inform process evaluation, giving it some similarities to Hybrid 1 which involves effectiveness studies with additional process evaluation. The PEDAL Trial did not involve interviews with administrators, policymakers, or other departments (e.g., Physiotherapy), however, which could have informed wider implementation. If planning the qualitative sub-study with an implementation mindset, these participants might have been included, giving further insights. This hybrid design is advocated in specific conditions, for example, where the intervention has face validitybase and minimal risk (1). A possible limitation to this approach is that the trial context itself may create barriers to implementation, as experienced within, a strong initial evidence the PEDAL Trial.

Hybrid 2 emphasizes clinical effectiveness and implementation more equally – for example, testing an intervention in “best” and “worst” and “medium” case conditions. This sounds appealing; however, it would be necessary to develop insights into what would make a case better or worse in relation to implementation, which may be an iterative process. For example, we can see retrospectively that some clinical sites involved in the PEDAL Trial had characteristics that might present more challenges to implementation, and it is unlikely that previously published studies would have given these insights.

The third Hybrid design involves supplementing an implementation study with data collection relating to intervention outcomes. This is advocated especially where it seems likely that the outcomes of the intervention will be heavily influenced by different and less controlled contexts. This seems likely to be the case for many rehabilitation interventions which involve behavior change for the participant, input from a multidisciplinary team, reorganization of the physical environment, and reallocation of resources. In the current financial context of the UK National Health Service many service changes must be made within existing resources. This is a complex challenge and is likely to require different strategies, such as co-production and change management.

### 4.4. Strengths and limitations

Study strengths include the quantity of qualitative data available for analysis. The concept of data saturation is more relevant to Grounded Theory, however, no new themes were emerging on completion of data collection and analysis. Two researchers cross-checked one another's interpretation of the data (CB, JS) and analysis continued to the point of developing novel explanatory theory. The qualitative sub-study included both racially and geographically diverse participants who were involved in the trial in multiple ways. We talked to people who were considered “drop-outs” from the intervention, which is unusual as people often leave the study and are not available further data collection. This added a further dimension to analysis.

Limitations included the challenges of data collection in busy clinical contexts, with background noise and interruptions. This made transcription harder but the researchers prioritized the needs of participants in relation to interview timing and location and this led to a high participation rate. Interviews varied substantially in length, which reflects differences in how reflective and communicative people were. The involvement of a second researcher for a minority of interviews may have introduced some inconsistencies and this risk was minimized where possible through training and supervision. There was a substantial time delay between participants consenting to the study and qualitative data collection taking place. This is because consent was given to participation in the whole study, of which the qualitative sub-study was only one stage which took place after people had experienced the intervention. Because of the delay, we ensured that people received an additional information letter by a study employee based in their site, just before the qualitative data collection was due to take place and were advised that they did not have to participate. When considering Provider participant numbers, it was not possible to calculate the percentage of providers who were interviewed relative to the total number of possible providers. Recruitment took place from the pool of all people providing or supporting care within the Hemodialysis Unit and PEDAL Trial team at different points in time. The total number of people employed within the Units fluctuated over time.

## 5. Conclusion

This paper reports on original insights from a large, rigorous qualitative process evaluation of a multi-center RCT which evaluated clinical effectiveness and cost effectiveness of a six-month intradialytic exercise programme when compared with usual care. The analysis has led to novel explanatory theory with relevance for evaluation of rehabilitation interventions. The “Ideal Scenario” is provided to guide trialists in pre-emptive and ongoing risk analysis relating to trial feasibility and long-term intervention implementation. This has international relevance as the detailed analysis led to identification of key aspects of different clinical contexts that were optimal and trialists can risk-assess their own clinical contexts in relation to these characteristics. Key insights include the need for careful integration of the trial within the clinical context to optimize promotion of the trial in the short-term and engagement and ownership of the intervention in the long-term. Strong leadership in both the clinical and trial teams is crucial to underpin a proactive and empowering culture.

The challenges of delivering an RCT of a complex rehabilitation intervention in a way that does not negatively impact potential for long-term implementation make it important to consider alternative study designs. Realist RCTs may provide more nuanced and informative results which indicate who can benefit from the intervention and in what circumstances. Effectiveness – Implementation Hybrid study designs prompt more careful consideration and integration of principles of Implementation Science research at an earlier stage in the research journey. This may help to counteract possible negative impacts of the trial experience on the clinical context that could jeopardize long-term implementation.

## Data Availability

The datasets generated and/or analyzed during the current study are not publicly available due the risks of identifiability through combined data but are available from the corresponding author on reasonable request supported by appropriate ethics review. Requests to access the datasets should be directed to cbulley@qmu.ac.uk.
