# Supplementary material for: Feasibility of randomized controlled trials and long-term implementation of interventions: Insights from a qualitative process evaluation of the PEDAL trial
Source: Front Rehabil Sci. 2023 Feb 1;4:1100084. doi: 10.3389/fresc.2023.1100084 (PMC9928991; doi:10.3389/fresc.2023.1100084)
Supplement: Supplementary file 1 [file Datasheet1.docx]

**Supplementary File 1: Topic guides**


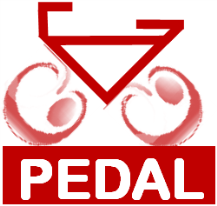


**PrEscription of intra-Dialytic exercise to improve quAlity of Life in patients with chronic kidney disease (The PEDAL TRIAL)**

**SERVICE PROVIDERS: Topic Guide**

- What is your name?
- Do any of you know each other already? How?
- What is your role in the renal unit?
- How did you first hear about the study?
  - What happened next?
- What did you expect initially?
- Is it what you expected?
- How did you feel about it / what did you think about that?
- What is your role in relation to the study?
- What does it involve?
- Has this changed over time?
- Does it have any impacts on you?
- Is there anything that makes your role harder?
- Is there anything that makes it easier?
- What do you think of the intervention?
- What do you think about physical activity / exercise more generally?
- What do you think that patients think / feel?
- What do you think that family or carers think / feel?
- In your view, what would the ideal scenario be?
- What is the most important think you would like us to know? Or What is the most important thing you think has been said today?
- Is there anything you would like to add privately?


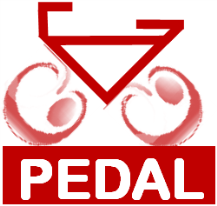


**PrEscription of intra-Dialytic exercise to improve quAlity of Life in patients with chronic kidney disease (The PEDAL TRIAL)**

**STUDY PARTICIPANTS: Topic guide**

- What is your name?
- Do any of you know each other already? How?
- How did you hear about the study?
- Why did you decide to join the study? What made you say ‘yes’?
  - What happened next?
- What did you expect initially?
- Is it what you expected?
- Has anything changed for you since you joined the study?
  - in relation to physical activity or exercise?
  - When you’re in the hospital?
  - When you’re outside the hospital?
  - Do you do anything differently?
  - Do you feel any differences?
  - Is there any difference in how you spend your time when you’re in the hospital?
  - Is there any difference in how you spend your time outside the hospital?
- What do you think about physical activity / exercise more generally?
- What is the renal unit like?
- How often / how long do you have to be (t)here?
- What influences the way you spend your time when you are in the renal unit?
  - Hospital staff?
  - Research staff?
- What influences the way you spend your time when you are outside the renal unit?
- Is there anything that makes it easier to be active?
- Is there anything that makes it harder to be active?
- What is the most important think you would like us to know? Or What is the most important thing you think has been said today?
- Is there anything you would like to add privately?
