# Supplementary figures and images for: Feasibility of randomized controlled trials and long-term implementation of interventions: Insights from a qualitative process evaluation of the PEDAL trial
Source: Front Rehabil Sci. 2023 Feb 1;4:1100084. doi: 10.3389/fresc.2023.1100084 (PMC9928991; doi:10.3389/fresc.2023.1100084)

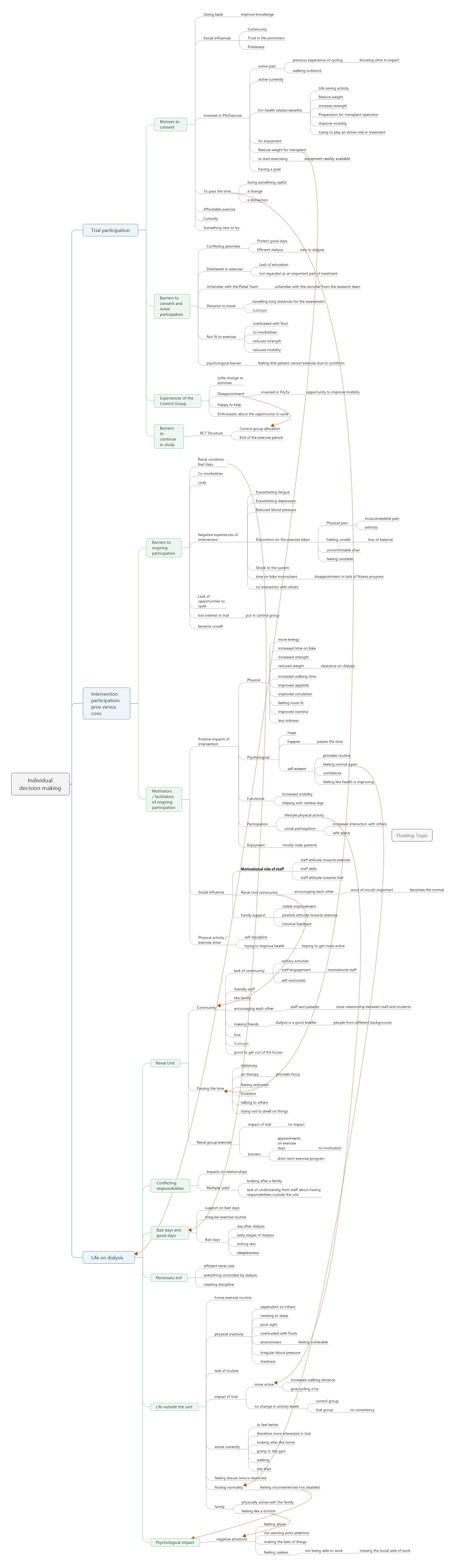

Supplement: Supplementary file 2 [file Image1.jpeg]

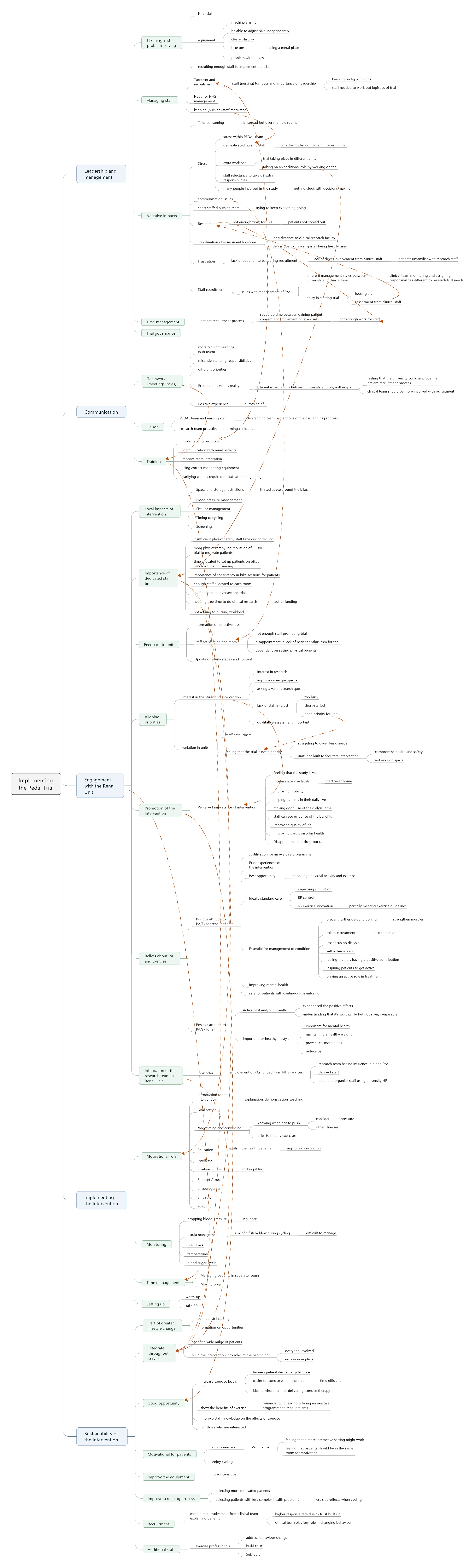

Supplement: Supplementary file 3 [file Image2.jpeg]
